# Neuropsychological profiles of patients suffering from hallucinogen persisting perception disorder (HPPD): A comparative analysis with psychedelic-using and non-using controls

**DOI:** 10.1038/s41598-024-82216-x

**Published:** 2024-12-31

**Authors:** Georg Leistenschneider, Tomislav Majić, Simon Reiche, Thomas G. Riemer

**Affiliations:** 1 Department of Psychology, Humboldt-Universität zu Berlin, 12489 Berlin, Germany; 2 Campus Charité Mitte, Charité – Universitätsmedizin Berlin, corporate member of Freie Universität Berlin and Humboldt-Universität zu Berlin, Psychiatric University Clinic at Hospital St. Hedwig, 10115 Berlin, Germany; 3 Institute of Clinical Pharmacology and Toxicology, Charité - Universitätsmedizin Berlin, corporate member of Freie Universität Berlin, Humboldt-Universität zu Berlin, and Berlin Institute of Health, 10115 Berlin, Germany

**Keywords:** Psychiatric disorders, Eye manifestations, Experimental models of disease, Diagnosis, Health care

## Abstract

Classic psychedelics like LSD and psilocybin are showing promising effects in treating certain psychiatric disorders. Despite their low toxicity and lack of an addictive potential, in some individuals, psychedelics can be associated with persisting psychological harms. Hallucinogen Persisting Perception Disorder (HPPD) is one of those complications, a rare disorder characterized by enduring perceptual symptoms without impaired reality control. While the phenomenological aspects of HPPD have been characterized, the neuropsychological consequences have remained understudied. This study probes the neuropsychological profiles of eight individuals with HPPD, utilizing a comprehensive test battery. Performance is benchmarked against normative data and compared with two control groups, each comprising eight matched subjects—with and without prior psychedelic use. The assessment of individual performances revealed below average results in tests of visual memory and executive function in some subjects. No significant differences were observed in alpha-adjusted comparisons with controls, whereas unadjusted analyses were suggestive of impaired executive functions among HPPD patients. Together, these preliminary results underline the need for further focused research into the neuropsychological dimensions of HPPD.

## Introduction

Hallucinogen Persisting Perception Disorder (HPPD) is a complex condition characterized by the persistence of perceptual symptoms following the use of certain groups of psychoactive substances^1^. By definition, HPPD is associated with having ingested hallucinogens, i.e., especially classic psychedelics (`psychedelics´) like lysergic acid diethylamide (LSD) and psilocybin^2^. Despite being a rare disorder, HPPD significantly impacts the well-being of affected individuals, with persistent perceptual disturbances that can lead to distress, impaired daily functioning, and reduced quality of life^3,4^. Given the growing body of evidence for the use of psychedelics as therapeutics^5^and the increasing prevalence of their recreational use^6^, it is crucial to thoroughly understand the intricacies of this disorder in order to foster effective preventive and harm reduction strategies for the management of serious side effects^7^.

The Diagnostic Manual of Mental Disorders, Fifth Edition (DSM-5) defines HPPD as the re-experiencing of one or more perceptual symptoms originally encountered during intoxication with hallucinogens^8^. While the DSM-5 initially delineated perceptual example symptoms based on a study by Abraham in 1983 (*n* = 123)^9^, a recent review of case reports by Vis et al. in 2021 (*n* = 97)^10^ has suggested a broader spectrum of associated perceptual disturbances.

In academic discussions regarding the acute and post-acute effects of psychedelic substances, the term “hallucination” is frequently employed. However, it is noteworthy to underscore that per definition, in HPPD, reality control is maintained, with the individuals retaining awareness of their non-ordinary perceptions. Consequently, a more precise term for these perceptual anomalies is “pseudo-hallucinations”, aligning with clinical terminology and conceptual clarity^11^. This distinction also indicates that the term “hallucinogen” is misleading, especially in the context of HPPD.

Despite its impact on individuals, the prevalence of HPPD is somewhat elusive due to a scarcity of epidemiological studies. A web-based study (*n *= 2,455)^4^ estimated the overall prevalence of HPPD among psychedelic users to be 4.2%, with 10.5% being female and a mean age of 21.6 years (*SD *= 3.7), which has been the foundation for the prevalence estimates reported in DSM-5. However, this study is not without its limitations, including reliance on self-reported data, the predominantly Western composition of the sample, with requirement for internet access, and potential selection bias stemming from the exclusive nature of participant recruitment, potentially favoring individuals with specific experiences to report. Additionally, differentiating HPPD from other medical conditions and mental disorders further complicates epidemiological investigations^8,12^.

Although the development of HPPD is most often associated with consumption of classic psychedelics, it can also emerge after use of other `consciousness-expanding´ substances, such as MDMA^13^, ketamine^10^, and cannabinoids^1,14^. Of note, there have even been few reports of patients experiencing symptoms of HPPD after use of non-hallucinogenic substances, such as amphetamines^15^and risperidone^16^.

Regarding the pathophysiology of HPPD, current neurobiological hypotheses suggest disturbances in visual pathways as underlying etiopathology of the disorder^15^. In specific, chronic disinhibition possibly through a reduced GABAergic output has been proposed as biochemical mechanism^17^, whereas impaired function of crucial visual areas, such as the lateral geniculate nucleus, has been proposed on a structural level^2,18^.

Some authors have suggested that HPPD could as well be a functional disorder^19^. As to date no biological correlates for the disorder have been identified, a functional etiopathogenesis cannot be ruled out. However, unlike psychosis or anxiety-related symptoms persisting during the post-acute period of the psychedelic drug action, HPPD displays phenomenological overlaps with neurological disorders of the brain, like epileptic aura phenomena, migraine, or encephalitis^13,20^. However, more research is needed to investigate if functional aspects might as well contribute to the genesis of the disorder.

There is a growing body of evidence indicating that psychedelics can be associated with post-acute effects in different dimensions, including mood, perception of self and others, and cognitive flexibility^21,22^. Specifically, acute psychedelic experiences have been associated with impaired attention, memory and executive functions^23^.

In contrast, post-acute changes of executive functions have been reported for some substances of the psychedelic group, including improvements for ayahuasca and deterioration for LSD^24^. Given that HPPD might be considered as a disorder which reflects post-acute persisting effects of psychedelics at least on the dimension of Visionary Restructuralization^25^, it appears to be crucial to investigate if changes in cognitive functions also persist in the post-acute period^26–28^. However, while the general phenomenology of HPPD has been reasonably characterized, the neuropsychological consequences of this disorder remain largely unexplored. A recent review^1^reported that so far, only five neuropsychological case assessments have been conducted on HPPD, with limited conclusions due to the lack of a standardized set of tests^11,29–31^.

Our study represents the first attempt to systematically assess the cognitive performance of HPPD patients, both at the individual level using normative data and by comparing them with two healthy control groups: one group consisting of non-symptomatic psychedelic users, and another group consisting of psychedelic-naïve participants. Employing an explorative cross-sectional design, a comprehensive neuropsychological battery was administered covering major cognitive domains, aiming to estimate the cognitive implications of HPPD.

## Methods

This project is part of the larger LZESH (German acronym for *long-term effects of serotonergic hallucinogens*) study. The comprehensive findings will be published elsewhere (Reiche et al., *manuscript in preparation*). LZESH is an observational study aimed at examining the psychopathological and neuropsychological long-term consequences of serotonergic psychedelic consumption. The project includes individuals aged 18–50 years who have either had at least one lifetime encounter with a serotonergic psychedelic (e.g. LSD, psilocybin, dimethyltryptamines, or mescaline) or have had no prior experience with such substances.

While the LZESH main study focuses on otherwise healthy subjects, this sub-study investigates individuals suffering from HPPD, who were recruited in addition to the main cohort. The use of cannabis was permitted without restrictions in both the HPPD and the non-HPPD samples. In addition, participants were required to have an Alcohol Use Disorders Identification Test score of ≤ 14. Strict thresholds were imposed on the lifetime history of other psychotropic substances in the non-HPPD group (maximum lifetime use of other illegal substances such as opioids, amphetamines, or cocaine < 10 experiences (for each drug); < 20 experiences for methylenedioxymethamphetamine; one experience is equal to one occasion of drug consumption, even if there was a second helping (the colloquial term would be “trip”). However, due to the high prevalence of patients with HPPD having a history of psychotropic drugs other than psychedelics and cannabis, it was not feasible to maintain these thresholds in the HPPD group.

This study was conducted with consideration to the Strengthening the Reporting of Observational Studies in Epidemiology (STROBE) guidelines^32^. Ethical approval was obtained from the Ethics Committee of Charité Universitätsmedizin Berlin (Approval No. EA4/203/17). Written informed consent was secured from all participants, and the procedures involving human subjects were guided by the ethical principles of the 1964 Declaration of Helsinki and its subsequent amendments.

### Sample

For this subset of the study, eight participants (1 female) with a mean age of *M* = 27.6 years (*SD *= 5.6) diagnosed with HPPD using the diagnostic criteria of the DSM-5^8^ were recruited. Using age, gender, education years, lifetime cannabis use, and lifetime alcohol use as covariates, the participants were then matched to two control groups. The first control group consisted of individuals with lifetime psychedelics use serving as an additional matching criterion (psychedelic-using control). This approach was adopted to delineate the specific cognitive implications of HPPD while effectively controlling for the confounding variable of prior psychedelics use.

The second control group encompassed individuals who had abstained from psychedelics use (non-using control). This control group was designed to closely resemble a portion of the general population that matched the HPPD group demographically. It allowed for an assessment of the effects of HPPD in conjunction with prior psychedelic substance use.

### Measures

Sociodemographic information, including age, gender, and educational level, as well as medical history and lifetime use of psychotropic substances, and psychopathological features such as depressive symptoms were collected through questionnaires. The diagnoses of HPPD were made by experienced psychiatrists according to the DSM-5 criteria^8^, while other major psychiatric disorders as well as neurological conditions were determined through clinical evaluation. As to date, there is no validated instrument available assessing HPPD symptoms, we employed a self-designed tool to collect data on the reported visual disturbances. This instrument assessed the frequency and duration of each symptom. The selection of items was based on a symptom list by Baggott et al. (2011). A team of psychologists and medical students under the supervision of an experienced neuropsychologist assessed the neuropsychological performance through a comprehensive test battery.

The assessment included tests that measured a wide range of cognitive abilities including Wechsler Adult Intelligence Scale 4^th^ edition (WAIS IV) digit-symbol and symbol search subtests; Wechsler memory scale 3^rd^ edition revised (WMS IIIR) digit span forwards and backwards; Wechsler memory Scale 4^th^ edition (WMS IV, German Version) logical memory with immediate and delayed recall trials; Rey-Osterrieth complex figure (ROCF) test with copy, immediate recall, and delayed recall trials; Stroop test (German Version); trail making test (TMT); Wisconsin card sorting test 64-item version (WCST-64); Testbatterie zur Aufmerksamkeitspruefung Version 2.2 (TAP, test battery for the assessment of attention) with subtests for alertness and divided attention (a*ssessed on a fujitsu computers siemens amilo L1310G, intel celeron M 380 1* × *1.6 GHz, ATI Radeon Xpress 200 M—128 MB VRAM, Display: 15.40 inch 16:10, 1280* × *800 pixels, running Windows XP home edition*); Tower of London (ToL), Regensburger word fluency test (RWT). A brief description of each assessment can be found in Table 1. References to each neuropsychological test are provided in the supplements *(Supplementary Table S1*).

**Table 1:** List of all used assessments including brief description of task.

Test	Sub-test	Task
Attention functions		
Wechsler Adult Intelligence Scale IV (WAIS IV)	Digit Symbol	Participant must match numbers with specific symbols within a set time limit
	Symbol Search	Participant must identify whether a target symbol appears within a group of symbols within a set time limit
Testbatterie zur Aufmerksamkeitsprüfung (*engl.: Testbattery for Attention Testing*, TAP)	Alertness (phasic, tonic)	Participant responds to visual stimuli by pressing a button as quickly as possible (with and without a warning sound)
	Divided attention	Participant responds to both visual and auditory stimuli simultaneously
Stroop Test (German Version)		Participant reads out colour names, then names coloured bars and finally names the ink colour of colour words which do not correspond to the actual word while ignoring the word’s meaning, all as quickly as possible
Trail Making Test (TMT-A)	Test A	Participant connects numbered circles in ascending order
Visual perception		
Rey-Osterrieth Complex Figure Test (ROCF)	Copy	Participant reproduces a complex geometric figure from observation
Memory		
ROCF	Recall, delayed recall	Participant reproduces the complex geometric figure from memory
Wechsler Memory Scale III R (WMS IIIR)	Digit span forwards/ backwards	Participant repeats digit spans from memory forwards and backwards
Wechsler Memory Scale IV (WMS IV: German Version)	Logical Memory	Participant repeats details of two short stories immediately and after a 20 min timespan
Executive functioning		
Tower of London (ToL; German Version)		Participant moves colored balls between pegs to recreate a target configuration while following specific rules
Regensburger Wortflüssigkeitstest (*engl.: Regensburg Test of Word* *Fluency,* RWT)	cat. fluency/ cat. change	Participant generates as many words as possible within a specific category or changing categories
Wisconsin Card Sorting Test 64 Items (WCST-64)		Participant matches cards based on changing rules
Trail Making Test (TMT-B)	Test B	Participant connects alternating numbered and lettered circles in ascending order

To evaluate the individual performance of participants in the HPPD group, their test scores were transformed into *z*-scores or percentile ranks (*PR*) relative to reference samples detailed in the respective test manuals. Scores falling below a *z*-score of –1 or a *PR* of 16 were classified as below average. This approach is informed by the neuropsychological criteria for identifying possible deficits in mild cognitive impairment (MCI), as outlined by Jak et al.^33^. MCI was chosen as an orientation because it represents the mildest clinically relevant level of cognitive impairment. Importantly, these thresholds were merely adopted and are not intended to imply that participants meet the diagnostic criteria for this condition.

### Statistical analysis

The matching procedure was employed to compare the HPPD cases with appropriate control groups and ensure covariate balance. To achieve optimal balance between the HPPD and control data, various matching strategies, such as Nearest Neighbour Matching, Optimal Pair Matching, and Optimal Full Matching, were evaluated based on recommendations from Greifer^34^. Propensity scores were estimated through logistic regression, and parameters including low Standardised Pair Difference, eCDF statistics, and variance ratios close to one^35^ were used to assess balance quality. The Optimal Pair Matching method demonstrated the most suitable parameters and was selected as the preferred approach for matching in this study.

The statistical analysis and matching procedure were conducted using RStudio version 2023.03.0 + 386^36^, R version 4.2.3, and the MatchIt software package for R, version 4.5.1^37^. A demographic comparison was performed between the matched control and HPPD groups to examine sample characteristics. Additionally, an unmatched comparative analysis was conducted between the two control groups. For these comparisons, two-sided Welch *t*-test were employed to assess variables such as age, education years, lifetime use of psychedelics (in experiences), integration of psychedelic experiences into daily life, lifetime use of cannabis (in experiences), lifetime alcohol intoxications (estimated as > 4 standard drinks or > 40 g alcohol), use of other illicit substances during the lifetime (in experiences), lifetime history of psychiatric treatment, measures of fluid and crystalline intelligence, i.e. Raven’s Standard Progressive Matrices (SPM)^38^and Wortschatztest (WST)^39^, and the German language and simplified version of the Beck Depression Inventory (BDI-V)^40^.

To compare performance on each neuropsychological test, two-sided Mann Whitney *U* Tests were used due to the assumption of non-standard normal distribution and the non-directional nature of the research question. A significance level of *p *< 0.05 was employed to determine statistical significance, indicating that the results were significantly different from what would be expected under the null hypothesis. To control for potential type I errors, the false discovery rate was controlled using the Benjamini–Hochberg procedure for multiple comparisons^41^. Given the novel and exploratory nature of the project and the field of research in general, unadjusted alpha values were included in the analysis to explore potential associations, bearing in mind the possibility of false-positive interpretations. In the results, adjusted *p*-values are marked with the suffix *a *(*p*_*a*_).

## Results

### Clinical visual characteristics of the HPPD group

**Fig. 1:**
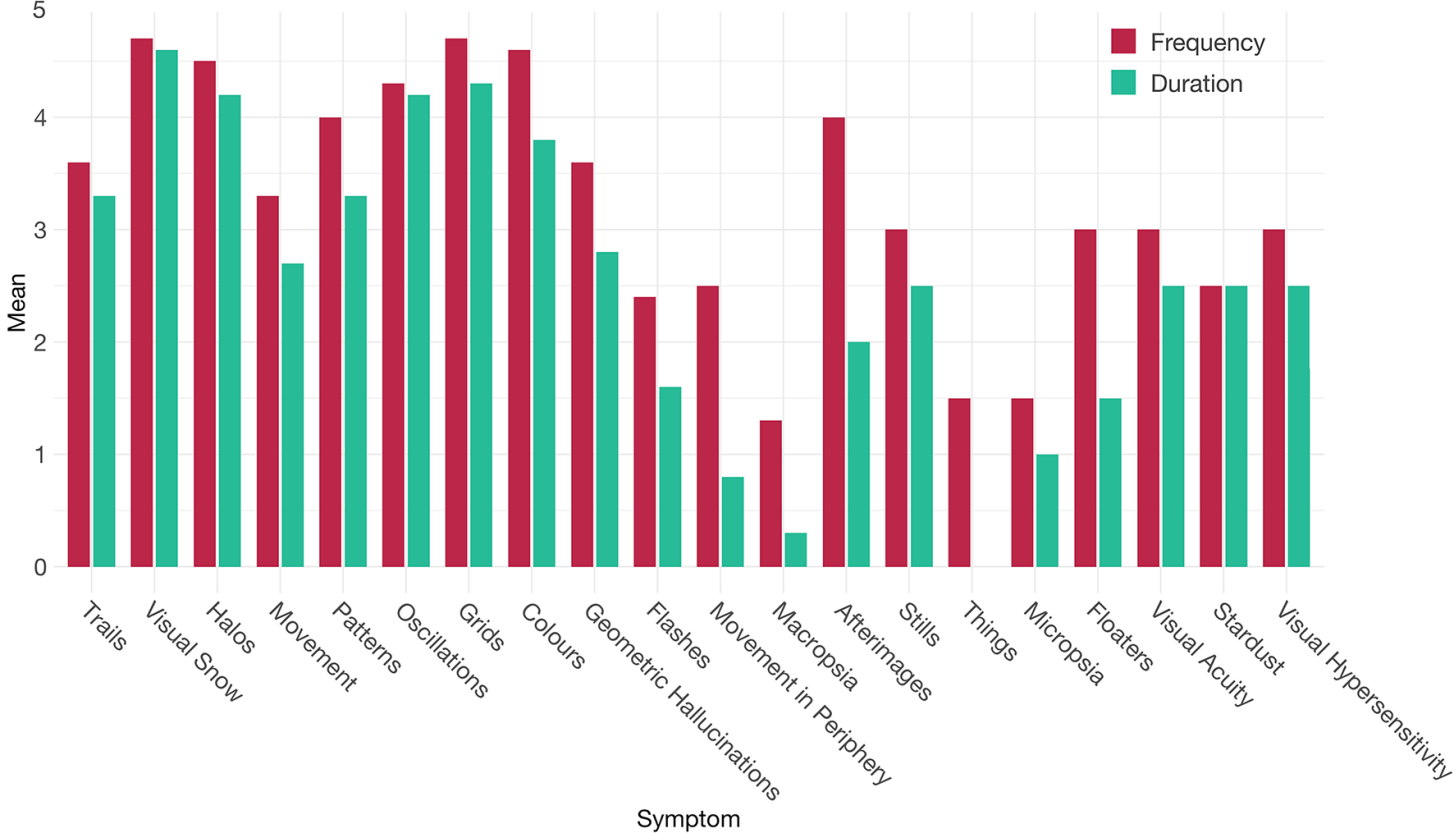
Frequency and Duration of Reported Visual Symptoms. Overview of visual symptoms reported by two or more patients, sorted by the number of reports from left to right, with the most reported symptoms first. For those experiencing a given symptom, occurrence frequency was assessed on a five-point Likert scale, ranging from 0 (never) to 5 (more than once per hour). Symptom duration varied from 0 (a few seconds) to 5 (constant).

The majority of HPPD patients (*n* = 7) reported constant or almost constant occurrences of visual snow (view covered by television-like static) and trails (stationary afterimages following moving objects). Other frequently reported symptoms included halos (circles of light around bright light sources or objects [*n* = 6]), movement (perception of stationary objects appearing to grow, shrink, or breathe [*n* = 6]), patterns (patterns or structures appear on surfaces [*n* = 6]), oscillations (increased irritation of oscillations or bright light sources [*n* = 6]), and grids (increased irritation of grid or net-like patterns [*n* = 6]). A complete list of visual symptoms reported by at least two patients is given in Table 2. In addition, Fig. 1 shows frequencies and durations of the perceived visual HPPD symptoms.

**Table 2:** Common visual symptoms reported in the HPPD sample.

Symptom	Description	Reported by *n* participants
Halos	Circles of light around objects, resembling a halo	6
Movement	Stationary objects appear to move, breathe, grow, or shrink	6
Stills	Moving objects appear to be stationary	2
Trails	Moving objects leave trailing images behind	7
Colors	Colors appear more vivid or intense	5
Patterns	Seeing patterns or surface textures that aren’t there	6
Things	Seeing objects or entities that aren’t there	2
Oscillations	Oscillations or flashes of light, similar to those on TV or in neon colors, are more disturbing than usual	6
Grids	Mesh or grid structures, or closely spaced lines, are more disturbing than usual	6
Geometric Hallucinations	Seeing stationary or moving geometric shapes	5
False Perception of Movements in the Peripheral Fields	Seeing movements in the periphery of your field of vision from objects that aren’t really there	4
Flashes	Seeing flashes of light even though no source is present	5
Micropsia	Living beings, objects, or entities in your surroundings appear smaller	2
Macropsia	Living beings, objects, or entities in your surroundings appear larger	3
Visual Snow	Seeing particle movements like fine snow, "static," or TV white noise	7
Stardust	Tiny luminous specks become visible within the visual field	2
Visual Acuity	Vision is subjectively experienced as more acute	2
Visual Hypersensitivity	Visual stimuli are more prone to causing discomfort or annoyance	2
Floaters	Apparent floaters in the visual field due to particles in the vitreous of the eye	2
Afterimages	A vivid visual afterimage persists when the eyes shift focus or are closed	3

### Demographic data analysis

Due to the rigorous matching procedure, all of the matched covariates yielded insignificant differences (Table 3). With an average education exceeding 16 years, the sample demonstrates a high level of education. The HPPD group and the psychedelic-using control group exhibited very similar patterns of psychedelic use, with the majority of individuals in both groups having used LSD and none having used ayahuasca*.* Neither group had consumed psychedelics within the last three months. The usual setting for psychedelic use was similar in both groups, however only the psychedelic-using control participants reported use in a spiritual setting, whereas only the HPPD group reported settings such as “in nature” in the “other” category and “at home”. The HPPD group reported a numerically larger number of other illicit substance uses than the control groups, although the difference was not statistically significant. Furthermore, the HPPD group did not statistically differ from the control groups in terms of intelligence (SPM and WST) and depressive symptoms (BDI-V). However, the HPPD group exhibited descriptively lower scores in the assessment of intelligence and higher burden of depressive symptoms compared to the controls.

**Table 3:** Demographical characteristics of sample compared utilising Welch’s t-test (*n* = 16).

	HPPD *M(SD)* (*n* = 8)	Psychedelic-using control *M (SD)* (*n* = 8)	Non-using control *M(SD)* (*n* = 8)	*t*-score (*df)* *p-*value HPPD vs. psychedelic-using control	*t*-score (*df)* *p-*value HPPD vs. non-using control
Age	27.6 (5.6)	29.4 (4.2)	24.9 (3.8)	−0.704 (13.036) *p* = 0.494	1.153 (12.237) *p* = 0.2711
Gender (female) n (%)	12.5	12.5	12.5	-	-
Education (years)	16.1 (2.0)	16.1 (1.9)	16.7 (1.8)	0 (13.981) *p* = 1.0	−0.593 (13.777) *p* = 0.563
Lifetime use of psy.*	26.9 (22.1)	29.9 (41.5)	0 (0)	−0.177 (10.659) *p* = 0.863	-
Psychedelics used (in %)*					
LSD	100	75	-		
LSA	12.5	25	-		
Psilocybin	75	62.5	-		
Ayahuasca	0	0	-		
DMT	25	25	-		
5-MeO-DMT	0	25	-		
Mescaline	0	25	-		
DOM, DOI, DOB	0	0	-		
2C-E, 2C-B	50	0	-		
* Other*	25	0	-		
Setting (in %)					
Party	25	12.5	-		
Spiritual	0	25	-		
Therapeutic	25	37.5	-		
No special setting	25	25	-		
Other	25	0	-		
Lifetime use of cannabis*	366.3 (407.6)	573.4 (919.0)	123.3 (125.9)	−0.583 (9.652) *p* = 0.573	1.611 (8.325) *p* = 0.144
Lifetime amount of alcohol intoxications**	221.1 (189.7)	201.4 (174.6)	205.2 (156.0)	0.216 (13.905) *p* = 0.832	0.183 (13.497) *p* = 0.858
Lifetime use of other illicit substances*	236.8 (485.9)	14.8 (11.0)	1.4 (2.9)	1.292 (7.007) *p* = 0.237	1.370 (7.001) *p* = 0.213
Lifetime history of psychiatric treatment (1 = yes, 0 = no)	0.6 (0.5)	0.8 (0.5)	0.3 (0.5)	−0.509 (13.829) *p* = 0.619	1.528 (13.829) *p* = 0.149
Ravens Standard Progressive Matrices	49.9 (6.2)	54.6 (1.4)	54.9 (4.6)	−2.107 (7.715) *p* = 0.070	−1.822 (12.951) *p* = 0.092
Wortschatztest***	30.1 (12.3)	37.0 (1.6)	35.6 (2.1)	−1.571 (7.239) *p* = 0.159	−1.249 (7.423) *p* = 0.250
BDI-V	29.9 (16.7)	21.3 (10.2)	19.3 (18.5)	1.245 (11.579) *p* = 0.238	1.206 (13.865) *p* = 0.248

*use is measured in experiences (colloquial “trips”), ** > 4 standard drinks or > 40 g alcohol, ***N = 15 (7 HPPD cases assessed).

An examination of the demographic data within the two control groups revealed notable disparities in age, lifetime consumption of psychedelics and other prohibited substances, as well as on the history of psychiatric interventions (*Supplementary Table S2*).

### Assessment of individual neuropsychological performance in the HPPD group using normative data

The test scores were subjected to a comparative analysis against normative data derived from multiple standardization studies to assess the potential clinical relevance of cognitive implications. Detailed data points and normative scores are provided in the *Supplementary Table S3*. The findings indicated no clear impairment, as most test scores fell within the range of expected norms.

However, notable observations emerged in the immediate and/or delayed recall component of the ROCF test in five of the participants with subpar performance relative to the normative population. Furthermore, four of the eight participants in the HPPD group showed below average performance in the completion of the TMT-B task. The scores depicted a range of subpar *z*-scores spanning from *z* = −1.07 to *z* = −1.87. Finally, the performance in category completion and total correct items in the WCST fell below the average range for three subjects, with rankings ranging between the 2nd and 5th percentiles.

### Comparison between the HPPD group and the non-using control group

The results of the neuropsychological assessment of the HPPD group and the non-using control group are summarised in Table 4. After adjusting for type I errors, no significant differences were detected.

**Table 4:** Comparison of neuropsychological test scores between HPPD group and non-using controls utilising Mann Whitney *U* Test (*n* = 16, 95% CI) and FDR alpha adjustment.

	HPPD *M(SD)* (*n* = 8)	Non-using control *M(SD)* (*n* = 8)	HPPD vs. non-using control	Adjusted *p*-values
WAIS IV				
Digit-symbol	61.1 (6.9)	72.6 (10.0)	***W* = 8, *p* = 0.013**	*p*_*a*_ = 0.346
Symbol search	39.1 (4.8)	44.9 (7.9)	*W* = 16.5, *p* = 0.114	*p*_*a*_ = 0.591
WMS IIIR				
Digit span, forwards	9.1 (1.6)	8.8 (1.5)	*W* = 38.5, *p* = 0.517	*p*_*a*_ = 0.795
Digit span, backwards	8.1 (2.0)	8.6 (2.3)	*W* = 28, *p* = 0.711	*p*_*a*_ = 0.888
WMS IV				
Logical memory-immediate recall	17.6 (2.2)	18.3 (2.2)	*W* = 27, *p* = 0.634	*p*_*a*_ = 0.867
Logical memory-delayed recall	15.2 (2.8)	16.1 (3.4)	*W* = 25, *p* = 0.493	*p*_*a*_ = 0.795
Rey-Osterrieth Complex Figure*				
Figure copy	35.5 (0.5)	34.1 (2.0)	*W* = 42, *p* = 0.099	*p*_*a*_ = 0.591
Immediate recall	21.1 (5.1)	19.6 (7.0)	*W* = 35, *p* = 0.451	*p*_*a*_ = 0.795
Delayed recall	19.9 (6.8)	20.4 (6.5)	*W* = 26.5, *p* = 0.908	*p*_*a*_ = 0.983
Stroop Test				
Word reading time (s)	28 (2.7)	26.4 (2.1)	*W* = 44, *p* = 0.204	*p*_*a*_ = 0.795
Colour naming time (s)	45.5 (5.1)	42.9 (3.7)	*W* = 42.5, *p* = 0.291	*p*_*a*_ = 0.795
Interference time (s)	64 (8.9)	58.9 (4.7)	*W* = 42, *p* = 0.317	*p*_*a*_ = 0.795
Trail Making Test				
Trail A time (s)	26.9 (11.1)	28.9 (6.9)	*W* = 24, *p* = 0.431	*p*_*a*_ = 0.795
Trail B time (s)	68.8 (24.4)	46.3 (9.9)	*W* = 49, *p* = 0.083	*p*_*a*_ = 0.591
WCST (64 Items)*				
Total correct	47.4 (8.8)	49.9 (10.4)	*W* = 22, *p* = 0.520	*p*_*a*_ = 0.795
Perseverative errors	6.8 (3.8)	7.3 (4.3)	*W* = 25.5, *p* = 0.815	*p*_*a*_ = 0.921
Non-perseverative errors	10.6 (8.6)	7.6 (6.2)	*W* = 33, *p* = 0.600	*p*_*a*_ = 0.866
Categories completed	3 (2.1)	3.9 (2.0)	*W* = 22, *p* = 0.454	*p*_*a*_ = 0.795
TAP, alertness				
Reaction time without warning (ms)	244 (17.5)	248.5 (19.8)	*W* = 23.5, *p* = 0.400	*p*_*a*_ = 0.795
Reaction time with warning (ms)	242.8 (15.7)	242.3 (23.5)	*W* = 32, *p* = 0.958	*p*_*a*_ = 0.996
TAP, divided attention				
Auditory reaction time (ms)	529 (68.7)	571.5 (68.3)	*W* = 22, *p* = 0.328	*p*_*a*_ = 0.795
Visual reaction time (ms)	749.1 (55.5)	721 (32.2)	*W* = 42, *p* = 0.328	*p*_*a*_ = 0.795
**Tower of London**	17.5 (1.5)	17.3 (1.7)	*W* = 35.5, *p* = 0.740	*p*_*a*_ = 0.888
Regensburger Word Fluency				
Semantic category	23.5 (6.1)	29.3 (6.3)	*W* = 15.5, *p* = 0.092	*p*_*a*_ = 0.591
Categorical switching	16 (3.3)	15.5 (2.9)	*W* = 35.5, *p* = 0.751	*p*_*a*_ = 0.888

*N = 15 (7 non-using controls assessed), bold values = significant results, _*a*_ *p*-values after alpha adjustment.

In the unadjusted comparison, the performance of the non-using control group (*M* = 72.6 (*SD* = 10.0)) in the WAIS IV Digit-symbol subtest demonstrated higher scores compared to the HPPD case group (*M* = 61.1 (*SD* = 6.9); *W* = 8, *p* = 0.013, *p*_*a*_ = 0.346). No differences were detected in unadjusted comparisons of other neuropsychological measures.

### Comparison between HPPD group and psychedelic-using control group

Table 5 presents the cognitive performance comparison between the HPPD group and the psychedelic-using control group. Following alpha adjustment, again no significant differences were observed between these groups. Prior, to alpha adjustment, disparities were found in two measures:

**Table 5:** Comparison of neuropsychological test scores between HPPD group and psychedelic-using controls utilising Mann Whitney *U* Test (*n* = 16, 95% CI) and FDR alpha adjustment.

	HPPD *M(SD)* (*N* = 8)	Psychedelic-using-control *M(SD)* (*N* = 8)	HPPD vs. psychedelic-using control	Adjusted *p*-values
WAIS IV				
Digit-symbol	61.1 (6.9)	64.6 (7.0)	*W* = 24.5, *p* = 0.461,	*p*_*a*_ = 0.705
Symbol search	39.1 (4.8)	44 (3.6)	*W* = 13*, p* = 0.051	*p*_*a*_ = 0.372
WMS IIIR				
Digit span, forwards	9.1 (1.6)	8.1 (1.6)	*W* = 44, *p* = 0.217	*p*_*a*_ = 0.625
Digit span, backwards	8.1 (2.0)	7.9 (2.2)	*W* = 35, *p* = 0.789	*p*_*a*_ = 0.894
WMS IV				
Logical memory-immediate recall	17.6 (2.2)	17.3 (2.7)	*W* = 35, *p* = 0.791	*p*_*a*_ = 0.894
Logical memory-delayed recall	15.2 (2.8)	16.1 (2.7)	*W* = 25.5, *p* = 0.525	*p*_*a*_ = 0.758
Rey-Osterrieth Complex Figure*				
Figure copy	35.5 (0.5)	33.8 (2.6)	*W* = 32, *p* = 0.293	*p*_*a*_ = 0.689
Immediate recall	21.1 (5.1)	21.5 (4.3)	*W* = 22.5, *p* = 0.900	*p*_*a*_ = 0.916
Delayed recall	19.9 (6.8)	21.2 (4.9)	*W* = 21, *p* = 0.746	*p*_*a*_ = 0.894
Stroop Test				
Word reading time (s)	28 (2.7)	26.5 (3.0)	*W* = 40.5, *p* = 0.396	*p*_*a*_ = 0.689
Colour naming time (s)	45.5 (5.1)	40.6 (1.6)	*W* = 49, *p* = 0.081	*p*_*a*_ = 0.422
Interference time (s)	64 (8.9)	57.3 (6.2)	*W* = 46.5, *p* = 0.14	*p*_*a*_ = 0.524
Trail Making Test				
Trail A time (s)	26.9 (11.1)	24.3 (6.9)	*W* = 34, *p* = 0.875	*p*_*a*_ = 0.916
Trail B time (s)	68.8 (24.4)	43.8 (6.4)	***W* = 52, *p* = 0.040**	*p*_*a*_ = 0.372
WCST (64 Items)				
Total correct	47.4 (8.8)	54.6 (3.4)	*W* = *13.5, p* = *0.057*	*p*_*a*_ = 0.372
Perseverative errors	6.8 (3.8)	5.8 (2.0)	*W* = 33.5, *p* = 0.916	*p*_*a*_ = 0.916
Non-perseverative errors	10.6 (8.6)	3.6 (2.1)	***W* = 51.5, *p* = 0.044**	*p*_*a*_ = 0.372
Categories completed	3 (2.1)	4.6 (0.7)	*W* = 20, *p* = 0.161	*p*_*a*_ = 0.524
TAP, alertness				
Reaction time without warning (ms)	244 (17.5)	238.4 (17.6)	*W* = 36.5, *p* = 0.673	*p*_*a*_ = 0.894
Reaction time with warning (ms)	242.8 (15.7)	232.6 (17.1)	*W* = 40, *p* = 0.442	*p*_*a*_ = 0.705
TAP, divided attention				
Auditory reaction time (ms)	529 (68.7)	507.9 (74.4)	*W* = 41, *p* = 0.382	*p*_*a*_ = 0.689
Visual reaction time (ms)	749.1 (55.5)	749.4 (99.4)	*W* = 36, *p* = 0.713	*p*_*a*_ = 0.894
**Tower of London**	17.5 (1.5)	16.6 (1.6)	*W* = 42, *p*^*1*^ = 0.295	*p*_*a*_ = 0.689
Regensburger Word Fluency				
Semantic category	23.5 (6.1)	29.5 (7.2)	*W* = 18, *p* = 0.154	*p*_*a*_ = 0.524
Categorical switching	16 (3.3)	13.3 (5.0)	*W* = 40.5, *p* = 0.397	*p*_*a*_ = 0.689

*N = 14 (6 psychedelic-using controls), bold values= significant results, _*a*_* p-values after alpha adjustment*.

The TMT-B showed differences, with the HPPD group requiring more time to complete the task compared to the psychedelic-using control group (*W* = 52, *p* = 0.040, *p*_*a*_ = 0.372). The HPPD group required a mean duration of *M* = 68.8 (*SD* = 24.4) seconds, while the psychedelic-using control group solved the trail with a time of *M* = 43.8 (*SD* = 6.4) seconds.

Another difference emerged before alpha adjustment when examining the incidence of non-perseverative errors in the WCST between the HPPD group and the psychedelic-using control group (*W* = 51.5, *p* = 0.044, *p*_*a*_ = 0.372). While participants in the HPPD group made an average of *M* = 10.6 (*SD* = 8.6) non-preservative errors, the psychedelic-using control group recorded *M* = 3.6 (*SD* = 2.1) errors.

### Comparison between psychedelic-using control group and non-using control group

The analysis of neuropsychological performance between the control groups revealed no statistically significant distinctions among them (*Supplementary Table S4).*

## Discussion

In our study investigating potential neuropsychological consequences of HPPD, we present the following observations:

(1) Evaluation against norm-referenced data from standardized tests revealed that the cognitive performance of the HPPD group was, mostly, within the normal range. However, closer examination on the subject level revealed that five individuals within the HPPD group exhibited below-average performance in the Rey-Osterrieth Complex Figure (ROCF) recall trial. This trial requires participants to memorize and later reproduce a complex geometric figure, both immediately after being presented, and after a 20-min delay. Such findings may imply deficits in visual-spatial memory or in cognitive functions related to memory and visual processing in a clinical setting. Furthermore, four out of the eight individuals in the HPPD group recorded completion times that were below the average on the Trail Making Test Part B (TMT-B), which involves connecting circles containing alternating numbers and letters in sequence. This result could suggest diminished cognitive flexibility and executive functioning capabilities. Lastly, three participants from the HPPD group showed inferior performance on certain tasks of the Wisconsin Card Sorting Test (WCST), where participants must discern and apply a hidden rule to match cards, further pointing to executive dysfunction.

(2) Statistical analyses comparing the HPPD group with the two control groups, after adjusting for alpha levels, found no significant differences. However, it is important to highlight that identifying significant differences was challenging due to the small sample size and the broad spectrum of neuropsychological tests utilized in this exploratory study, which necessitated the application of alpha error correction for multiple testing. Given the small sample size, the appropriateness of using standard inferential statistics is debatable, as they may not reliably identify differences unless the effect sizes are large. Nonetheless, these analyses may still offer indicative insights to be further explored in future studies.

In *unadjusted* comparisons, several differences came to light. It’s essential to note that these differences could potentially be the result of chance, considering the large number of tests performed. However, the pattern of these differences remains compelling, highlighting decrements in executive functioning domains. This partly aligns with the previously noted subpar performance in individual assessments when using normative data. While the negative results of the adjusted analysis prohibit definite statements on the neuropsychological impact of HPPD, the unadjusted findings may potentially serve as a starting point for more focused investigations in future studies. Therefore, they are now thoroughly examined to elucidate their potential implications:

The unadjusted comparison of the HPPD group with the non-using control group pointed towards lower performance in the digit symbol test. Originally considered a measure of complex attention, this test is now recognized for assessing a wide range of cognitive abilities, including attention, psychomotor speed, visual-perceptual functions, and executive functioning^42^.

In case the group difference is not due to factors unrelated to psychedelic use, diminished performance in this test could be attributed to either HPPD itself or being independent from HPPD, but related to past use of psychedelics. Despite all participants noting no usage of psychedelics in the past three months, cognitive long-term influences are plausible even in the absence of perceptual symptoms. A recent meta-analysis revealed that various psychedelics might have distinct post-acute neuropsychological consequences, with LSD being linked with decreased executive performance and ayahuasca being linked to increased executive performance^24^. Notably, the majority of the HPPD patients reported using LSD and none ayahuasca. However, the performance of the digit symbol test in psychedelic-users without HPPD, which also reported use of LSD, did not differ from that of the non-using group, making the use of LSD as the sole explanation unlikely.

When further comparing the HPPD group to the psychedelic-using control group prior to alpha adjustment, additional differences hinting towards a possibly lower performance of the HPPD group emerged in the TMT-B and the WCST. These findings could add towards a potential executive deficit specific to HPPD.

An alternative interpretation of a decreased performance in the individuals with HPPD compared to the normative group as well as the HPPD group’s lower performance compared to the control groups in unadjusted analyses could lie in the predominantly visual nature of HPPD. Given that the mentioned neuropsychological tests prominently feature a visual component, there is a plausible argument that impaired visual functioning could exert an influence on the test performance of individuals with HPPD. Although current visual symptom load was not actively assessed in the testing sessions, three participants spontaneously noted difficulties during the assessment, due to ongoing symptoms. Furthermore, most of the participants had reported constant or nearly constant visual symptoms during the assessment of HPPD symptom burden. The persistence of visual disturbances in HPPD may affect tasks that rely heavily on visual perception and processing, as proposed by the below average performance in the ROCF in several participants. However, it should be noted that the tests in question are not the sole assessments in the battery reliant on unimpaired visual functioning. Considering the extent of any existing visual impairments, it would be expected that performance decrements would be consistently evident in assessments relying on visual functioning. Further research should strive to provide an objective characterization of those possible visual impairments.

There is a growing body of evidence suggesting that psychedelics may be promising therapeutic agents for the treatment of different psychiatric conditions, such as major depression^43^and substance use disorders^44^. Of note, increased cognitive flexibility following psychedelic experiences has been proposed as one major therapeutic mechanism of action for therapy with psychedelics^45^. Our findings from examining the neuropsychological profiles of individual participants in the HPPD group, as well as from unadjusted comparisons, could prompt extensive investigations into whether these effects are attenuated or even reversed in HPPD. However, while our study investigated recreational users with HPPD, controlled therapeutic settings differ significantly from recreational consumption for different reasons^46^: purity, quality and dosage of the substances can accurately be relied upon, the environment (`setting´) is specifically prepared for the use of psychedelics and the patients´ psychological condition (`set´) is rigorously monitored including the assessment of potential risk factors^47^.

Furthermore, dosing sessions are usually embedded in a series of drug-free therapeutic sessions, where patients are well-prepared for the experience, and potential post-acute adverse reactions can be sufficiently addressed^48^. Nevertheless, the risks of HPPD in the context of guided psychedelic therapy are still understudied^7^. One recent study reported persisting visual symptoms in 9.2% of healthy participants in studies employing LSD or psilocybin^49^, which all subsided within one week. To our knowledge, however, persisting perceptual and cognitive symptoms have been monitored only in some of the recent trials administering psychedelics in clinical populations. We tentatively suggest that the impact of HPPD should be investigated regarding the possible consequences on neuropsychological performance. If the presence of deficits were to be confirmed in future focused investigations, it could contribute to clarifying the neural underpinnings of HPPD*.*

Despite being the largest study conducted on the cognitive profile HPPD to date, recruitment of a satisfactory sample size remained challenging due to the overall low prevalence of HPPD. As a countermeasure, inclusion criteria had to be adapted, allowing for various amounts of illicit substance use in the HPPD group. However, this modification may have compromised the comparability of the HPPD and control groups. Importantly, the HPPD group exhibited a numerically higher frequency of other illicit substance use compared to the control groups, though these differences were not statistically significant. However, the comparatively high use may still have influenced cognitive performance. Notably, polydrug use has been shown to associate with impaired executive functions^50^. Furthermore, it is noteworthy that the HPPD patients in this study exhibited higher, although more variant levels of depressive symptoms and lower scores in the intelligence screening compared to both control groups. Those differences, however, did not reach statistical significance, either. Prior research has established a correlation between elevated depressive scores and lower intelligence with reduced executive performance^51^. This observed association within our sample does align with previous findings, suggesting that the presence of elevated depressive symptoms and lower intelligence scores may contribute to performance disparities in patients. However, it should be underlined that this might be inherent to the specific characteristics of our sample. Further investigations examining the demographic characteristics of HPPD patients are necessary to explore this in more detail.

Finally, the absence of neuropsychological assessment data preceding the onset of HPPD renders the design cross-sectional rather than longitudinal. This poses a significant challenge in neuropsychological research, given the lack of regular neuropsychological assessments among the general population, thereby obscuring baseline cognitive abilities. As a common practice, comparisons were made against normative population data to address this limitation.

To our best knowledge, this is the first study to date presenting a systematic assessment of cognitive functions in HPPD. Our sample, despite its small size, approximates the demographic characteristics of the large cohort study used to develop the DSM-5 criteria^4^, although the average age in our sample is descriptively 6 years older. Further strengths of the study embrace the inclusion of two different control groups (one psychedelic-using and one non-using), a matching procedure, and a comprehensive neuropsychological testing battery that covers all major cognitive domains. Attaining a suitable control sample in the realm of psychedelic research represents a notable challenge^24^. Nonetheless, the meticulous matching procedures employed in this study, combined with the norm-based appraisal of individual neuropsychological profiles, enabled us to gain a first impression of the possible neuropsychological sequelae of HPPD. Although this study did not demonstrate a significant difference between HPPD patients and matched control groups, the pattern of visual and executive deficits seen in individual HPPD patients as well as in the unadjusted comparison to the controls, could provide impetus for a more focused investigation with greater statistical power. Therefore, additional research is needed to further explore the neuropsychological implications of HPPD.

## Supplementary Information

Supplementary Information.

## Data Availability

The datasets generated during and/or analyzed during the current study are available from the corresponding author on reasonable request.
